# Two histologically colorectal carcinomas subsets from the serrated pathway show different methylome signatures and diagnostic biomarkers

**DOI:** 10.1186/s13148-018-0571-3

**Published:** 2018-11-09

**Authors:** José García-Solano, María C. Turpin, Daniel Torres-Moreno, Francisco Huertas-López, Anne Tuomisto, Markus J. Mäkinen, Ana Conesa, Pablo Conesa-Zamora

**Affiliations:** 1 Department of Pathology, Santa Lucía General University Hospital (HGUSL), C/Mezquita s/n, 30202 Cartagena, Spain; 2 Facultad de Ciencias de la Salud, Catholic University of Murcia (UCAM), Murcia, Spain; 3 Instituto Murciano de Investigaciones Biosanitarias (IMIB), Murcia, Spain; 4 Faculty of Experimental Sciences, Universidad Francisco de Vitoria, Carretera Pozuelo-Majadahonda km. 1.800 28223 Pozuelo de Alarcón, Madrid, Spain; 5 Microbiology and Cell Sciences Department, Institute for Food and Agricultural Sciences, University of Florida, Gainesville, FL USA; 6 Cancer and Translational Medicine Research Institute, Department of Pathology, and Medical Research Center, University of Oulu and Oulu University Hospital, Oulu, Finland; 7 Genomics of Gene Expression Laboratory, Centro de Investigación Príncipe Felipe (CIPF), Valencia, Spain; 8 Genetics Institute, Institute for Food and Agricultural Sciences, University of Florida, Gainesville, FL USA; 9 Department of Clinical Chemistry, Santa Lucía General University Hospital (HGUSL), C/Mezquita s/n, 30202 Cartagena, Spain

**Keywords:** Colorectal cancer, Methylome, Serrated, Immune response, Microsatellite instability, Epigenetics, Colon carcinogenesis, CD14, HLA-DOA

## Abstract

**Background:**

Altered methylation patterns are driving forces in colorectal carcinogenesis. The serrated adenocarcinoma (SAC) and sporadic colorectal carcinoma showing histological and molecular features of microsatellite instability (hmMSI-H) are two endpoints of the so-called serrated pathological route sharing some characteristics but displaying a totally different immune response and clinical outcome. However, there are no studies comparing the methylome of these two subtypes of colorectal carcinomas. The methylation status of 450,000 CpG sites using the Infinium Human Methylation 450 BeadChip array was investigated in 48 colorectal specimens, including 39 SACs and 9 matched hmMSI-H.

**Results:**

Microarray data comparing SAC and hmMSI-H showed an enrichment in functions related to morphogenesis, neurogenesis, cytoskeleton, metabolism, vesicle transport and immune response and also significant differential methylation of 1540 genes, including CD14 and HLA-DOA which were more methylated in hmMSI-H than in SAC and subsequently validated at the CpG, mRNA and protein level using pyrosequencing, quantitative polymerase chain reaction (qPCR) and immunohistochemistry.

**Conclusions:**

These results demonstrate particular epigenetic regulation patterns in SAC which may help to define key molecules responsible for the characteristic weak immune response of SAC and identify potential targets for treating SAC, which lacks molecular targeted therapy.

**Electronic supplementary material:**

The online version of this article (10.1186/s13148-018-0571-3) contains supplementary material, which is available to authorized users.

## Background

Serrated polyp pathway is considered as an alternative pathological sequence to the so-called adenoma-carcinoma sequence which is typically characterized by chromosomal instability and by ending up in the development of conventional carcinoma (CC) [1]. Less is known about the CRCs developed mainly in proximal colon through the serrated pathway, although high-level of microsatellite instability (MSI-H), BRAF mutation and CpG island methylation phenotype (CIMP) seems to be the driven forces in this carcinogenic process. The CRC showing histological and molecular features of MSI-H (hmMSI-H) [2, 3] is considered as one endpoint of the serrated route as well as serrated adenocarcinoma (SAC) which has a typical serrated morphology and remnants of serrated polyps (SP) adjacent to the invasive tumour [4, 5]. SAC has been recognized in the latest WHO classification of tumours of the digestive system as a new subtype of colorectal cancer (CRC) [6], accounting for 7.5 to 8.7% of all CRCs [5, 7] approximately one third of serrated pathway CRCs [8] and most are microsatellite stable (MSS) and can be either BRAF or KRAS mutated [9–11]. Criteria for SAC histologic diagnosis have been proposed [7] and recently validated in a series of 85 cases, and it has been shown to have a worse prognosis than conventional carcinoma (CC) [5]. Accordingly, SAC displays a higher frequency of adverse histological features at the invasive front including high-grade tumour budding and cytoplasmic pseudofragments, infiltrating growth pattern and weak peritumoural lymphocyte response [12]. Besides, mRNA microarray studies have demonstrated that SAC has a different expression profile compared to CC [13, 14]. Despite these features, there are no studies assessing the differences in the molecular signatures of SAC and the typical hmMSI-H nor specific markers nor the clinical differences between these two CRC subtypes.

Based on an initial histological evaluation, we aimed in this work to investigate the following issues:

-To discern which are the differentially methylated functions between SAC and hmMSI-H and if these could explain the histologic characteristics of these two entities

-To identify and validate at different levels those differentially methylated genes so they could be used as diagnostic markers or potential therapeutic targets

## Results

**Fig. 1:**
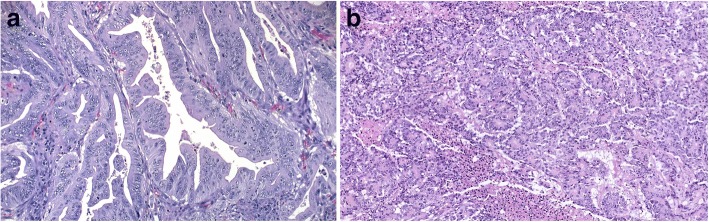
Histological morphology of colorectal cancer subtypes (H-E staining). **a** Serrated adenocarcinoma (SAC) showing serrated glands with large, rounded nuclei with prominent nucleoli and ample eosinophilic cytoplasm. Original magnification × 20. **b** Colorectal carcinoma showing histological and molecular features of microsatellite instability (hmMSI-H). The image represents a microglandular monomorphic pattern with a small area of necrosis. Original magnification × 15

The clinico-pathological features of the patients have been previously reported [5, 10] and are shown in Table 1. SACs were diagnosed on the basis of criteria proposed by Mäkinen et al. [7] and hmMSI-H according to prior established criteria [2] (Fig. 1). No significant differences were observed for confounding variables between SAC and hmMSI-H in the training and validation series (Table 1).

**Table 1:** Clinico-pathological features of the study cases

	Training set			DNA validation set (pyrosequencing)			RNA validation set (qPCR)			Protein validation set (IHC)		
	SAC	hmMSI-H		SAC	hmMSI-H		SAC	hmMSI-H		SAC	hmMSI-H	
	*n* = 39 (%)	*n* = 9 (%)	*p* value	*n* = 16 (%)	*n* = 9 (%)	*p* value	*n* = 19 (%)	*n* = 22 (%)	*p* value	*n* = 26 (%)	*n* = 21 (%)	*p* value
Gender												
Female	20 (51.3)	7 (77.8)		8 (50)	7 (77.8)		15 (78.9)	14 (63.6)		19 (73.1)	12 (57.1)	
Male	19 (48.7)	2 (22.2)	0.284	8 (50)	2 (22.2)	0.174	4 (21.1)	8 (36.4)	0.234	7 (26.9)	9 (42.9)	0.201
Age [SD]	71.6 [10.0]	66.4 [15.22]	0.094	72.0 [9.5]	70.3 [8.4]	0.267	69.0 [8.43]	69.75 [13.9]	0.839	69.9 [6.8]	70 [10.7]	0.969
Location												
Proximal	26 (66.7)	8 (100)		9 (56.3)	8 (88.9)		10 (52.6)	15 (68.2)		14 (53.8)	13 (61.9)	
Distal/rectum	13 (33.3)	1 (0)	0.182	7 (43.8)	1 (11.1)	0.107	9 (47.4)	7 (31.8)	0.309	12 (46.2)	8 (38.1)	0.399
Dukes’ stage												
A	4 (10.3)	1 (11.1)		2 (12.5)	1 (11.1)		3 (15.8)	6 (27.3)		4 (15.4)	4 (19.0)	
B	13 (33.3)	5 (55.6)		7 (43.8)	5 (55.6)		7 (36.8)	9 (40.9)		9 (34.6)	10 (47.6)	
C	17 (43.6)	3 (33.3)	0.846	7 (43.8)	3 (33.3)	0.846	9 (47.4)	7 (31.8)	0.522	13 (50.0)	7 (33.3)	0.523
WHO grade												
High	5 (12.8)	0 (0)		1 (6.3)	0 (0)		1 (5.3)	0 (0)		0 (0)	0 (0)	
Low	34 (87.2)	9 (100)	0.596	15 (93.8)	9 (100)	0.64	18 (94.7)	22 (100)	0.463	26 (100)	21 (100)	NA
Type												
Non-mucinous	34 (87.1)	6 (66.7)		14 (87.5)	6 (66.7)		17 (89.5)	19 (86.4)		22 (84.6)	18 (85.7)	
Mucinous	5 (12.8)	3 (33.3)	0.321	2 (12.5)	3 (33.3)	0.23	2 (10.5)	3 (13.6)	0.572	4 (15.4)	3 (14.3)	0.623

*SAC* serrated adenocarcinoma, *hmMSI-H* colorectal carcinoma showing histological and molecular features of microsatellite instability, *qPCR* quantitative PCR, *IHC* immunohistochemistry, *SD* standard deviation

### Differentially methylated functions

Bioinformatic analysis revealed a considerable number of GO biological processes (BP) differentially methylated in SAC vs. hmMSI-H: 40 GO terms obtained using ClusterProfiler while 76 terms where found using FatiGo (Additional file 1: Table S1). In general, differentially methylated genes were enriched for GO-BP terms related with biosynthesis (GO:0034654, GO:0031326, GO:0044271, GO:0018130); nitrogen and nucleic acid metabolism (GO:0090304, GO:0060255, GO:0051173, GO:0006807); RNA activity and transcription regulation (GO:0006355, GO:0032774, GO:2001141); protein secretion (GO:0051051, GO:0051047), neurogenesis (GO:0051960, GO:0030182, GO:0022008, GO:0030900), morphogenesis (GO:0048598, GO:0048562, GO:0009887, GO:0016331), sensory perception (GO:0007608, GO:0050911, GO:0050906); cytoskeleton and cell movement (GO:0048870, GO:0016477, GO:0051674) and immune response (GO:0002250).

**Fig. 2:**
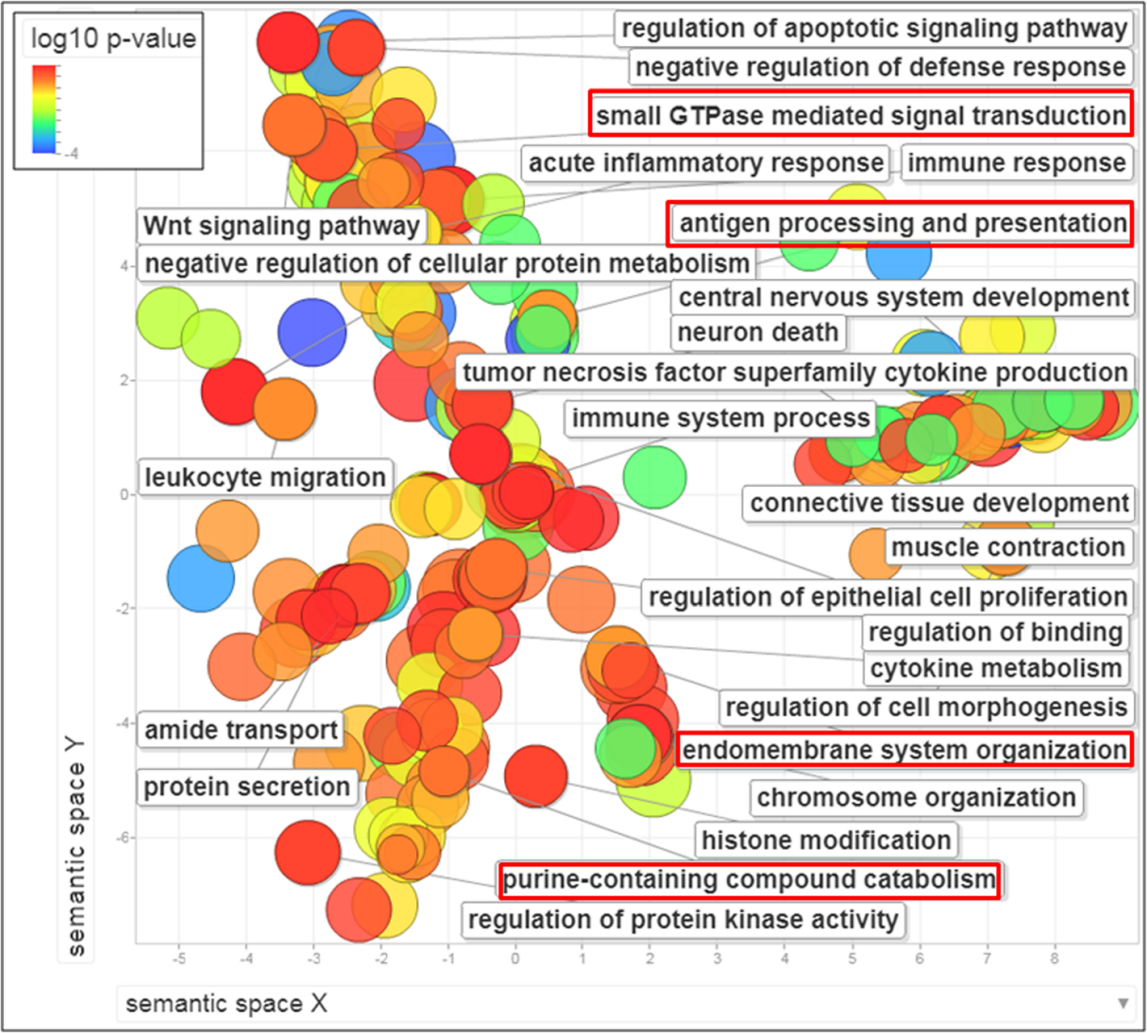
Terms of Gene Ontology biological processes enriched in the comparison between SAC and hmMSI-H for their methyloma profile. The scatterplot shows the biological processes which are globally differentially methylated between SAC and hmMSI-H after GO term redundancy reduction. The graph is represented in a two-dimensional space derived by applying multidimensional scaling to a matrix of the GO terms’ semantic similarities [35]. Terms associated with higher methylation in hmMSI-H are shown in red squares. Colour intensity indicates level of significance (log10 *p* value). Circle diameter indicates number of genes for each biological process

The scatterplot obtained using REVIGO shows the GO biological processes which are differentially methylated between SAC and hmMSI-H after the redundancy reduction (Fig. 2). GO cellular component and molecular function categories are shown as Additional file 2: Figure S1.

### Differentially methylated genes

The analysis of the methylome microarray data identified 1540 differentially methylated genes, 266 of which were more methylated in SAC than in hmMSI-H (Additional file 3: Table S2). No significant methylated genes were observed when comparing normal mucosa from SAC and normal hmMSI-H mucosa or when comparing Spanish and Finnish serrated tumour cases. Table 2 shows the list of the 42 most differentially methylated genes as obtained from the array analysis. Functions associated with these genes are shown in Additional file 4. Based on the extent of differential methylation grade, the importance of the biological functions, the design of suitable primers and the availability of antibodies, we decided to validate *HLADOA* and *CD14* at the DNA, mRNA and protein level. CD14 is a surface antigen, preferentially expressed on monocytes/macrophages that cooperate with other proteins to mediate the innate immune response to bacterial lipopolysaccharide. Alternative splicing results in multiple transcript variants (NCBI RefSeqs NM_000591.3, NM_001040021.2, NM_001174104.1, NM_001174105.1) encoding the same protein. HLA-DOA, in turn, is a member of the HLA class II which forms a heterodimer with HLA-DOB. This heterodimer, HLA-DO, is found in lysosomes in B cells and regulates HLA-DM-mediated peptide loading on MHC class II molecules.

**Table 2:** List of the 42 most differentially methylated genes (37 more methylated in hmMSI-H and 5 in SAC) as obtained from the array analysis

Gene symbol	>methyl. in	adj *p* val.	Gene name	Chr
*PDCD2L*	hmMSI-H	0.00101	Programmed cell death 2-like	19
*FGFR2*	SAC	0.00147	Fibroblast growth factor receptor 2	10
*CTSL2*	hmMSI-H	0.00159	Cathepsin L2	9
*TLR4*	hmMSI-H	0.00159	Toll-like receptor 4	9
*ANKRD10*	hmMSI-H	0.00350	Ankyrin repeat domain 10	13
*COPZ1*	hmMSI-H	0.00420	Coatomer protein complex, subunit zeta 1	12
*C17orf108*	SAC	0.00420	Chromosome 17 open reading frame 108	17
*CEP68*	hmMSI-H	0.00580	Centrosomal protein 68 kDa	2
*ZNF652*	hmMSI-H	0.00580	Zinc finger protein 652	17
*PCYOX1L*	hmMSI-H	0.00583	Prenylcysteine oxidase 1 like	5
*GALNT11*	hmMSI-H	0.00583	UDP-NADG:polypeptide N-acetylgalactosaminyltransferase 11	7
*MICAL2*	hmMSI-H	0.00583	Microtubule-associated monoxygenase, calponin and LIM domain 2	11
*OSCP1*	hmMSI-H	0.00583	Organic solute carrier partner 1	1
*ACAD10*	hmMSI-H	0.00583	Acyl-CoA dehydrogenase family, member 10	12
*MELK*	hmMSI-H	0.00583	Maternal embryonic leucine zipper kinase	9
***HLA-DOA***	hmMSI-H	0.00583	Major histocompatibility complex, class II, DO alpha	6
*KRT20*	hmMSI-H	0.00583	Keratin 20	17
*TMEM45B*	hmMSI-H	0.00583	Transmembrane protein 45B	11
*LOC285401*	SAC	0.00641	Uncharacterized LOC285401	3
*PPIL1*	hmMSI-H	0.00650	Peptidylprolyl isomerase (cyclophilin)-like 1	6
*CA13*	hmMSI-H	0.00709	Carbonic anhydrase XIII	8
*BCL7C*	hmMSI-H	0.00709	B cell CLL/lymphoma 7C	16
***CD14***	hmMSI-H	0.00850	CD14 molecule	5
*PLA2G4C*	hmMSI-H	0.00905	Phospholipase A2, group IVC (cytosolic, calcium-independent)	19
*LY6G6D*	hmMSI-H	0.00908	Lymphocyte antigen 6 complex, locus G6D	6
*TCF7L2*	hmMSI-H	0.00954	Transcription factor 7-like 2 (T-cell specific, HMG-box)	10
*RP11-165H20.1*	hmMSI-H	0.00954	CHIA-like pseudogene	1
*CHD6*	hmMSI-H	0.00954	Chromodomain helicase DNA-binding protein 6	20
*PARN*	hmMSI-H	0.00954	Poly(A)-specific ribonuclease	16
*KCNK15*	hmMSI-H	0.00954	Potassium channel, subfamily K, member 15	20
*TMEM209*	hmMSI-H	0.01015	Transmembrane protein 209	7
*GOLM1*	hmMSI-H	0.01016	Golgi membrane protein 1	9
*ARRDC1*	hmMSI-H	0.01016	Arrestin domain containing 1	9
*TRIM21*	hmMSI-H	0.01016	Tripartite motif containing 21	11
*PHAX*	hmMSI-H	0.01016	Phosphorylated adaptor for RNA export	5
*NMT2*	hmMSI-H	0.01016	N-myristoyltransferase 2	10
*ATP12A*	hmMSI-H	0.01016	ATPase, H+/K+ transporting, nongastric, alpha polypeptide	13
*ARHGAP30*	SAC	0.01016	Rho GTPase-activating protein 30	1
*WWP1*	hmMSI-H	0.01055	WW domain-containing E3 ubiquitin protein ligase 1	8
*GNAI3*	hmMSI-H	0.01055	Guanine nucleotide binding (G protein), α inhibit polypeptide 3	1
*CD48*	SAC	0.01055	CD48 molecule	1
*EXPH5*	hmMSI-H	0.01070	Exophilin 5	11

Genes chosen for validation by pyrosequencing, qPCR, and IHC are written in bold letters

*SAC* serrated adenocarcinoma, *hmMSI-H* colorectal carcinoma showing molecular and histological features of microsatellite instability

### Validation of methylated sites by pyrosequencing

The distribution of CpG islands in *HLA-DOA* and *CD14* enabled the design of primers to quantify the level of CpG methylation in these genes by pyrosequencing upon bisulfite-treated DNA. As shown in Table 3, the methylation percentage in *CD14* was generally lower than that in *HLA-DOA* CpG sites. Intriguingly, tumoural specimens from SAC, but not hmMSI-H cases, showed lower methylation level of *HLA-DOA* than normal adjacent samples. In contrast, CpG2 site from *CD14* displayed higher methylation in tumoural than in normal specimens from hmMSI-H patients, whereas no such significant difference was observed in SAC cases. Consistent with the microarray results, the percentage of CpG methylation in *HLA-DOA* and *CD14* was higher in hmMSI-H than in SAC at all CpG sites studied except for CpG3 in *HLA-DOA* (Table 3).

**Table 3:** Pyrosequencing for the relative quantitation of CpG methylation in *CD14* and *HLA-DOA* genes

Gene		*CD14*		*HLA-DOA*		
CpG site		CpG1	CpG2	CpG1	CpG2	CpG3
SAC-T (*n* = 16)	Mean	6.89	13.56	33.83	34.95	89.78
	SD	5.53	8.06	15.47	14.91	8.04
hMSI-H-T (*n* = 9)	Mean	13.64	22.38	50.94	48.12	94.61
	SD	6.15	8.9	14.81	13.62	2.36
SAC-T vs. hmMSI-H	*p* value	0.010	0.020	0.013	0.039	0.14
SAC-N (*n* = 8)	Mean	7.97	13.86	60.02	58.27	100
	SD	3.19	2.19	2.01	2.94	0
MSI-N (*n* = 6)	Mean	8.25	13.72	53.13	53.26	98.69
	SD	2.36	2.37	6.73	8.92	2.75
SAC-T vs. SAC-N	*p* value	0.616	0.911	0.0001	0.0003	0.006
hmMSI-H-T vs. hmMSI-H-N	*p* value	0.064	0.038	0.742	0.432	0.002
SAC-N vs hmMSI-N	*p* value	0.860	0.911	0.017	0.159	0.271

*SAC-T* tumoural tissue from serrated adenocarcinoma, *hmMSI-H-T* tumoural tissue from histological and molecular high-level microsatellite unstable colorectal carcinoma, *SAC-N* normal tissue adjacent to SAC, *hmMSI-H-N* normal tissue adjacent to hmMSI-H

### Validation by qPCR

**Fig. 3:**
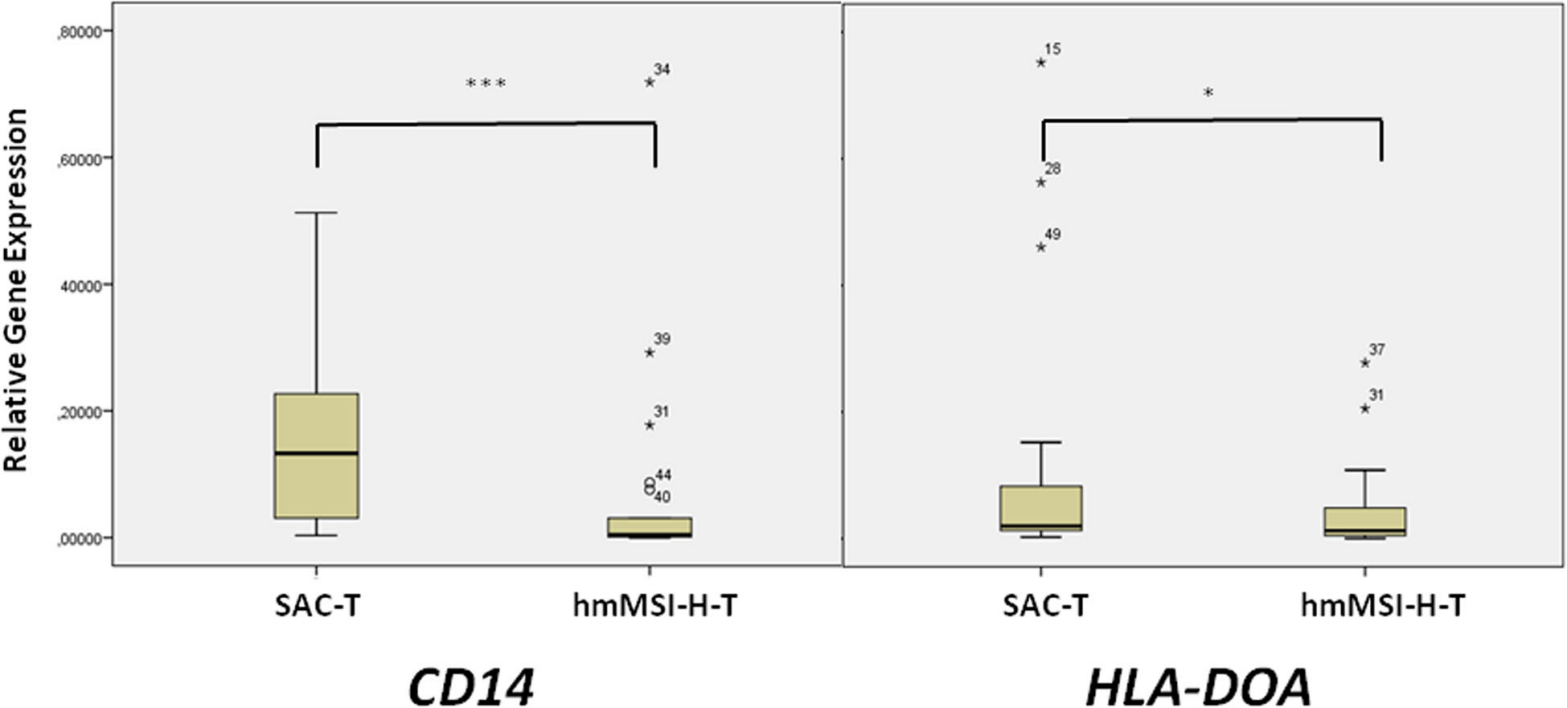
qPCR results of the mRNA expression of CD14 and HLA-DOA genes in SAC and CC tumoural tissue as well as in adjacent non-tumoural specimens. Asterisk indicates statistical significance.*p > 0.05; ***p > 0.005

With the aim of finding out whether higher CpG methylation in *HLA-DOA* and *CD14* in hmMSI-H correlates with a decreased expression of these genes, an analysis of the expression of *HLA-DOA* and *CD14* mRNA by quantitative PCR was performed. *HLA-DOA* expression did not show differences between tumoural and normal mucosa (median 0.00438 vs. 0.00467; *p* = 0.531), whereas CD14 expression was lower in tumoural than in normal mucosa, although not reaching statistical significance (median 0.0128 vs. 0.0599; *p* = 0.098). There were no differences when comparing tumoural and normal mucosa for each CRC subtype (Table 4). The qPCR results validated the microarray and pyrosequencing results as CRC cases with serrated histology showed higher expression of *CD14* (median 0.133 vs. 0.004; *p* = 0.004) and *HLA-DOA* (median 0.099 vs. 0.006; *p* = 0.047) than hmMSI-H cases, respectively (Fig. 3/Table 4).

**Table 4:** Quantitative analysis of the *CD14* and *HLA-DOA* mRNA expression in tumour and adjacent normal mucosa

Gene	Sample type	*N*	Mean ± SD	Median	IR
*CD14*	SAC-T	19	0.166 ± 0.16	0.133	0.509
	hmMSI-H-T	22	0.066 ± 0.16	0.004	0.718
	*p* value				0.004
	SAC-N	7	0.191 ± 0.17	0.198	0.386
	hmMSI-H-N	7	0.129 ± 0.24	0.037	0.667
	*p* value				0.383
*HLA-DOA*	SAC-T	19	0.0617 ± 0.11	0.099	0.375
	hmMSI-H-T	22	0.021 ± 0.03	0.006	0.138
	*p* value				0.047
	SAC-N	7	0.135 ± 0.01	0.0098	0.034
	hmMSI-H-N	7	0.07 ± 0.008	0.037	0.021
	*p* value				0.620

*SAC-T* tumoural tissue from serrated adenocarcinoma, *hmMSI-H-T* tumoural tissue from histological and molecular high-level microsatellite unstable colorectal carcinoma, *SAC-N* normal tissue adjacent to SAC, *hmMSI-H-N* normal tissue adjacent to hmMSI-H

### Validation by immunohistochemistry

**Fig. 4:**
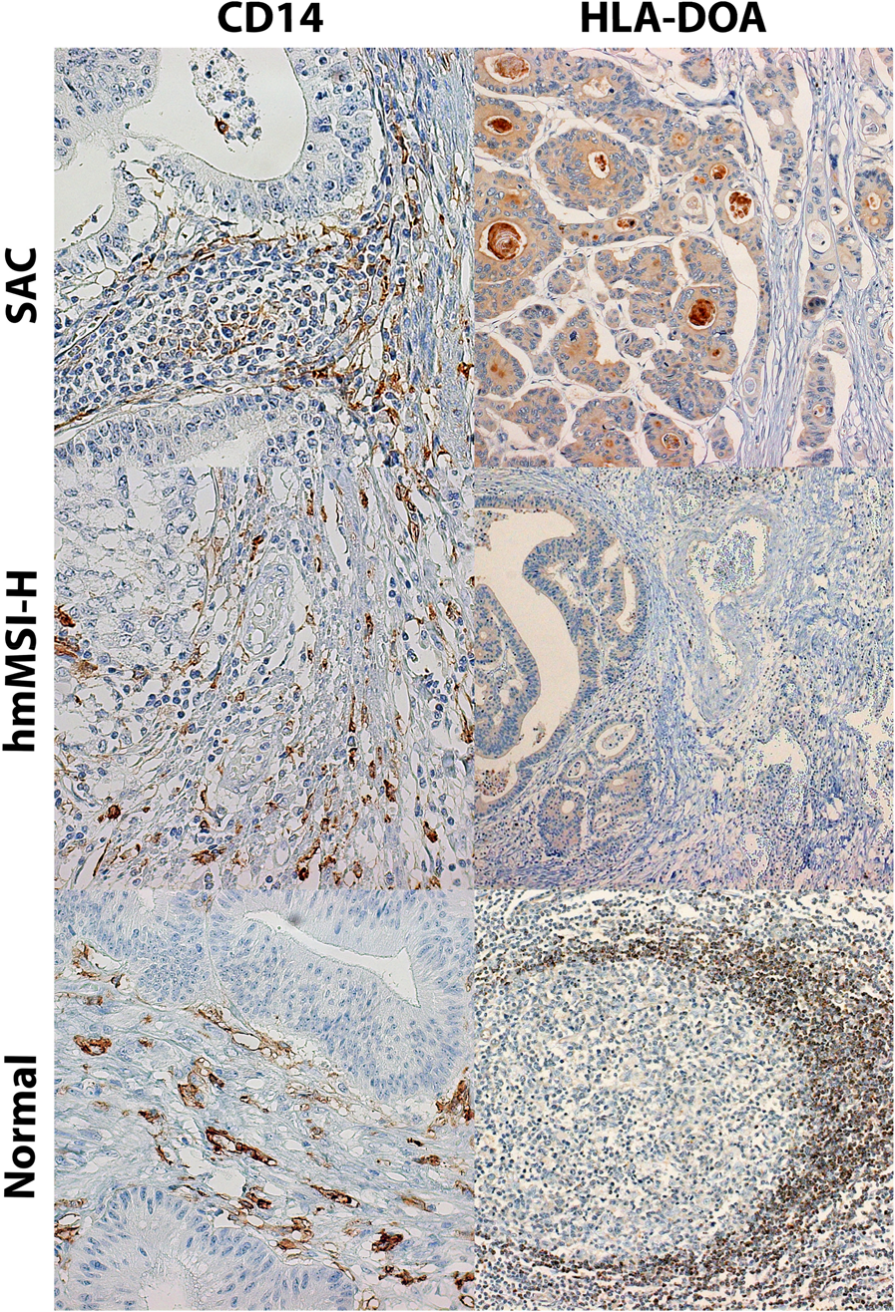
Immunohistochemical expression of CD14 and HLA-DOA in serrated adenocarcinoma (SAC) and CRC showing histological and molecular features of microsatellite instability (hmMSI-H) and in normal tissue (intestinal lamina propria and lymph node, respectively). Original × 40 magnification in all except in HLA-DOA in hmMSI-H (× 20)

In order to investigate whether differential methylated status of *HLA-DOA* and *CD14* in SAC compared to hmMSI-H could have an effect on protein expression within the tissue cells, immunohistochemistry for both HLA-DOA and CD14 was performed. Figure 4 shows the staining pattern for CD14 being cytoplasmic in stromal cells, not only in myeloid cells from monocyte/macrophague linage but also in some endothelial, (lower left image) whereas for HLA-DOA mantle zone cells from a lymph node were positive (lower right image). In SAC cases, a positive membranous staining for CD14 was observed in some stromal cells and lymphocytes with no expression in neoplastic glands. As shown in Fig. 4, the expression of CD14 was also absent in neoplastic glands from hmMSI-H cases and only few stromal cells stained positive and no staining was observed in lymphocytes. In tumoural cases, an intense cytoplasmic and luminal secretion staining for HLA-DOA was observed in SAC neoplastic glands whereas weak cytoplasmic expression considered as negative was observed in hmMSI-H tumour cells. In both cases, stromal cells were negative (Fig. 4).

Surprisingly, negative or weak expression of CD14 was statistically more frequent in SAC than in hmMSI-H (81.8% vs. 42.9%; *p* = 0.008) (Fig. 4; Table 5). As expected, moderate/strong HLA-DOA staining was higher in SAC than in hmMSI-H (30.8% vs 0%; *p* = 0.005), thus confirming array, pyrosequencing and qPCR results.

**Table 5:** Immunohistochemical expression of CD14 and HLA-DOA proteins

Protein	Sample type	*n*	Distribution (A–C) Intensity (1–3) score					Two-tier score		*p* value (Fisher)
			A1	A3	B2	B3	C3	Negative/weak (A1-B2)	Moderate/strong (B3-C3)	
CD14	SAC	26	11	10	0	3	2	21 (81.8)	5 (19.2)	
	hmMSI-H	21	3	6	0	5	*7*	9 (42.9)	12 (57.1)	0.008
HLA-DOA	SAC	26	9	8	1	8	0	18 (69.2)	8 (30.8)	
	hmMSI-H	21	17	4	0	0	0	21 (100)	0	0.005

Immunohistochemical markers in the tumours were evaluated by considering the distribution area score (A < one third, B = between one and two thirds, C = two-thirds-whole area) and the staining intensity (1 = none or weak staining, 2 = moderate, 3 = strong)

*SAC* serrated adenocarcinoma, *hmMSI-H-T* colorectal carcinoma with histological and molecular high-level microsatellite instability

### Association of CD14/HLA-DOA methylation with KRAS/BRAF/MSI-H status

In order to ascertain the relationship between the methylation of these genes with common biomarkers used in the clinical management of CRC, we performed an association study using the cases of DNA validation set. The results in Table 6 show that CD14 methylation is significantly associated with wild-type *KRAS*, V600E mutant *BRAF* and microsatellite instability. *HLA-DOA* methylation did not correlate with these markers except for a tendency with microsatellite instability (*p* = 0.069).

**Table 6:** Association of CD14 and HLA-DOA methylation (measured as percentage of methylated cytosine) with common colorectal carcinoma biomarkers (KRAS, BRAF, MSI)

Biomarker*		*CD14*			*HLA-DOA*		
		Median	IR	*p* value	Median	IR	*p* value
*KRAS*	Mutated	9.20	5.9		35.97	38.8	
	Wild-type	15.355	12	0.022	40.37	33.7	0.290
*BRAF*	Mutated	16.68	21.7		39.63	35.5	
	Wild-type	12.845	6.4	0.035	37.53	36.2	0.82
MSI	Unstable	16.7	19.1		40.95	30.2	
	Stable	11.32	6.1	0.001	31.9	39.6	0.069

*Codon 12 and 13 mutations in KRAS and V600E mutation in BRAF were considered. CpG site 2 for CD14 and CpG site 2 for HLA-DOA were considered. Similar results were obtained for site 1 (data not shown)

IR interquartile range, MSI microsatellite instability

## Discussion

A two-arm model has been proposed to explain the progression of the serrated pathway, the aberrant crypt foci-hyperplastic type being the earliest lesion which may develop into hyperplastic polyps and sessile serrated adenomas, or into traditional serrated adenomas, both of which may progress to CRC having serrated histology such as SAC and to CRC with histological and molecular features of microsatellite instability (hmMSI-H). Despite that these two CRC subtypes seem to share a common pathological route, the clinical manifestation and prognosis is surprisingly different. On the one hand, hmMSI is associated with an abundant immune response and good prognosis [15]. On the other hand, SAC has a worse outcome that conventional CRC as it displays a high frequency of histological adverse prognostic factors, the weak immune response being one of them, along with high tumour budding and infiltrative growth pattern. It is therefore important to study which molecular features distinguish SAC and hmMSI-H, not only for understanding the serrated polyp pathway but also for providing diagnostic markers and molecular targets for histology-based specific treatments. In this study, which is the first comparing the methylome of these two entities, we have observed important differences in the functions associated with methylated genes from each tumour type. First, most of these genes were more methylated in hmMSI-H than in SAC, thus suggesting that, in general, MSI-H development might more associated with aberrant hypermethylated, and thus silencing, genes than SAC. Given that differentially methylated genes were more methylated in SAC than in CC [16], SAC would show an intermediate hypermethylation status between hmMSI-H and CC. Functions differentially methylated are mostly related to metabolism, morphogenesis, neurogenesis, cytoskeleton and immune response. These findings are consistent with previous works analysing the expression profile of SAC highlighting the over-representation of these biological activities when compared to CC [13, 14]. Evasion of immune response has been proposed as an emerging hallmark of cancer, and in fact, weak peritumoural lymphocytic infiltration (PLI) is considered as one histological adverse prognostic factor [17, 18]. All this evidence is also consistent with the higher frequency of tumour infiltrating and peritumoural lymphocytes and “Crohn-like” inflammatory response found in hmMSI-H and the weak PLI and worse prognosis which characterize SAC [12]. This finding raises the question of which molecular factors determine these differences between these two CRCs. One possible explanation comes from the fact that hmMSI-H CRC, due to the dysfunction of the DNA mismatch repair machinery, generates a high number of neoantigens that stimulate the immune system whose effector cells are attracted to the tumour invasive front [19]. Given the allegedly common origin of hmMSI-H and SAC and some molecular similarities, one challenging therapeutic approach for SAC might be to make this CRC subtype recognizable for the immune system. In order to know which steps in the immune response could be affected, a list of significantly methylated genes has been obtained in this work most of them belonging to immune response.

From those significantly methylated genes, CD14 and HLADOA were chosen to be validated at the DNA, mRNA and protein level. CD14 is protein involved expressed mainly by monocyte/macrophage cell although some non-myeloid lineage cells such epithelial can also express it [20]. Apart from its role in response against sepsis, recent reports have found that CD14+ circulating monocytes and CD14+ tumour infiltrating macrophages are more frequently found in patients with CRC compared to healthy controls, these findings being associated with a higher plasma concentration of the immunosuppressive cytokine, IL10 [21]. These CD14+ monocytes are a subset of myeloid-derived suppressor cells (MDSC) which are key mediators in the negative regulation of immune responses [22]. Therefore, the observed methylation and CD14 gene silencing could have an immunostimulatory effect in MSI-H CRCs in contrast to SAC where *CD14* gene is less methylated and, subsequently, more expressed. Despite that our study validates *CD14* microarray result at the DNA and mRNA level, it was not able to confirm a lower expression of the CD14 protein in hmMSI-H. Possible reasons for that finding could be the expression of specific CD14 transcripts by myeloid cells or non-myeloid with other functions than immune tolerance or the fact that CD14 is also expressed by non-myeloid cells [20], thus making complicated its histological evaluation. Supporting the conflicting nature of this issue, some other studies have reported that CD14+ macrophages at the invasive front correlates with a more favourable prognosis in CRC patients with metastasis [23]. The expression of molecules expressed by immune cells such as CD14 will be dependent on the overall presence of these cells in the tumour microenvironment. One of our aims was to characterize molecules or immune cell types that are responsible for the difference observed between SAC and hmMSI-H. The higher *CD14* methylation and the decreased gene expression observed in hmMSI-H stress the point that, despite hmMSI-H having a remarkable immune response, some kind of difference in the CD14 function may exist between SAC and hmMSI-H.

Human leukocyte antigen (HLA) family members are antigen-presenting molecules which are expressed by virtually all cells in human body, including cancer cells, although the downregulation or aberrant expression of these molecules are used by the tumour to avoid immune response [24]. As regards to HLA-DOA, we validated the higher methylation and lower mRNA and protein expression in hmMSI-H compared to SAC. Very little is known about that protein as there are no studies linking it to colorectal carcinoma. Archer et al., by analysing the methylation status of 1505 CpG sites in hepatocellular carcinoma (HCC) tissue and comparing it with paired pre-neoplastic non-tumourous specimens from the same patients, observed that *HLA-DOA* was hypomethylated in tumoural tissue [25]. This finding supports a role of HLA-DOA, not only in HCC but in CRC tumour development. In agreement, our results in CRC also demonstrated a lower methylation in CRC compared to adjacent normal specimens, regardless the histological type of CRC. This observation did not reach statistical significance at the mRNA expression level, thus suggesting additional factors involved in the regulation of *HLA-DOA* expression. Very few articles correlated HLA-DOA with immune response features. Wang et al. reported that hypomethylation of CpGs in 6p21.3, where *HLA-DOA* is located, was associated with increased CD8 T cell tumour infiltration in serous ovarian cancer [26] whereas Ningappa et al. and Sindhi et al. demonstrated that HLA-DOA inhibits B cell presentation of antigen and consequently these authors proposed a potentially novel antirejection drug target [27, 28]. Our results are in line with these latter works; as transcription of major histocompatibility genes is silenced by DNA methylation [29] of upstream promoters, it would not be surprising that higher HLA-DOA methylation in hmMSI-H might have an effect on enhancing antigen presentation by B cells and generate a more prominent immune response.

## Conclusions

Our study identifies key functions and genes that might be important for understanding two CRC histological subtypes sharing a common pathological route but developing a dissimilar immune response and having a different clinical outcome. However, subsequent studies are needed to further characterize the interplay between the immune microenvironment and the tumour CRC cells in the serrated pathway.

## Material and methods

### Patients and tumour samples

SACs were diagnosed on the basis of criteria proposed by Mäkinen et al. (epithelial serrations, clear or eosinophilic cytoplasm, abundant cytoplasm, vesicular nuclei, absence of or less than 10% necrosis of the total surface area, mucin production and cell balls and papillary rods in mucinous areas of a tumour) [7]. hmMSI-H were diagnosed according to prior established criteria (mucinous, signet-ring cell, and medullary carcinoma, tumour infiltrating and peritumoural lymphocytes, “Crohn-like” inflammatory response, poor differentiation, tumour heterogeneity and “pushing” tumour border) (Fig. 1) [2]. Frozen samples of 21 and 18 SACs were retrieved from Santa Lucia General University Hospital (HGUSL), Cartagena, Spain, and Oulu University Hospital, Oulu, Finland, respectively. Additionally, nine matched hmMSI-H from HGUSL were included for the methylome microarray study. Validation by methylated sequences was performed on 16 Spanish SAC cases and nine hmMSI-H from the microarray subset. Validation by qPCR was performed upon frozen specimens of 12 SAC and nine hmMSI-H, and in addition, adjacent normal mucosa was also analysed from eight SACs and six hmMSI-H. Paraffin blocks of 26 SAC and 21 matched hmMSI-H, included in previous works, [10, 14] were used for immunohistochemistry (IHC) validation. The assessment of the MSI-H condition was confirmed at the molecular level as described previously by our group [10], and none of the hmMSI-H showed serrated morphology. The cases from DNA validation set were used to assess the correlation between gene methylation and KRAS, BRAF and MSI status. The study was approved by the Hospital Ethics Committee and was carried out in accordance with the ethical standards laid down in the 1964 Declaration of Helsinki and its later amendments. Written informed consent was obtained from all the patients.

### DNA extraction

A volume of approximately 10 mm^3^ was extracted from each frozen tissue using the disposable sterile biopsy punch. DNA was extracted following the manufacturer’s instructions (Qiagen, Hilden, Germany). Briefly, tissue was disrupted and homogenized in ATL buffer using a Tissueruptor (Qiagen), incubated with proteinase K and the homogenate was subjected to automatic DNA extraction using the Qiacube equipment and the QiaAmp DNA Mini Kit (cat no.:51306), both provided by Qiagen.

### Bisulfite treatment and DNA methylation assay

HumanMethylation450K BeadChip (Illumina, Inc., San Diego, CA), using Infinium HD Methylation assay for genome-wide DNA methylation screening, was employed. In brief, genomic DNA (1000 ng) from each sample was bisulfite converted with the EZ DNA Methylation Kit (Zymo Research, Orange, CA) according to the manufacturer’s recommendations. Bisulfite-treated DNA was isothermally amplified at 37 °C (20–24 h), and the DNA product was fragmented by an endpoint enzymatic process, then precipitated, resuspended, applied to an Infinium Human Methylation450K BeadChip (Illumina, San Diego, CA, USA), and hybridized at 48 °C (16–24 h). The fluorescently stained chip was imaged by the Illumina i-SCAN, and Illumina’s Genome Studio program (Methylation Module) was used to analyse BeadArray data to assign site-specific DNA methylation β-values to each CpG site. The data set supporting the results of this article are available in the GEO repository, GSE68060 in https://www.ncbi.nlm.nih.gov/geo/query/acc.cgi?acc=GSE68060.

### Preprocessing of methylation data

Processing of raw data was done using R packages. Probes with a low detection *p* value (*p* < 0.01) in more than 95% of the samples and those measuring SNPs or mapping in X or Y chromosomes were removed and normalization followed a three-step procedure. Firstly, a colour bias adjustment was applied using the methylumi R-package [30]. Then, wateRmelon [31] R-package was used to perform between-sample normalization by equalization of type I and type II backgrounds followed by separated quantile normalization of methylated and unmethylated intensities. Finally, A BMIQ [32] intra-sample normalization procedure, included in the wateRmelon R-package, was applied to correct the bias of type II probe values.

### Differential methylation functional profiling

The analysis of differentially methylated genes was performed using limma [33] R-package. Data were fitted to a linear model, and differential methylated genes were identified by using the empirical Bayes method included in the package. If the comparison was done between paired samples, a moderated paired *t* test was applied. A FDR-corrected *p* value of 0.05 was used as the threshold to select differentially methylated genes. Functional profiling of the differentially methylated genes was performed using ClusterProfiler and the FatiScan method included in the Babelomics [33, 34] web suite. For functional annotation, the Biological Process Database from Gene Ontology (GO) (www.geneontology.org) was used. Differentially methylated GO biological process was represented as scatterplot using REVIGO online package [35].

### Pyrosequencing

DNA methylation percentages of five different CpG island sites included in the microarray (two in CD14, three in HLA-DOA) were analysed and quantified by pyrosequencing. Bisulfite-converted DNA was previously amplified by PCR using Hot-Start GoTaq polymerase (Promega, Madison, WI) under the following conditions: 1 ul of DNA, 4 ul of 5X polymerase buffer, 0.2 mM dNTPs, 0.6 mM MgCl2, 0.3 μM of either biotin-labelled forward or reverse primers and 0.05 U/μl Hot-start Go Taq Flexi polymerase (Promega). PCR protocol was performed as follows: initial denaturation at 94 °C for 2 min, 35 cycles of 94 °C 10 s, 64 °C (CD14) or 60 °C (HLA-DOA) 10 s and 72 °C 50 s and a final extension step of 72 °C 7 min. Details of amplicon and primer sequences are provided in Additional file 5: Table S3. PCR products were verified using the QIAxcel DNA high-resolution electrophoresis system. Pyrosequencing of methylated sites was performed using the PyroMark Q24 (Qiagen) according to the manufacturer’s protocol. The methylation level was assessed using the PyroMark Q24 2.0.6 Software (Qiagen) by which the methylation percentage (mC/mC+C) for each CpG was calculated. The results are presented as the percentage (mean ± SD) of the different CpG sites studied for each of the CpG sites analysed whose sequences and relative positions are also shown as Additional file 5: Table S3.

### Quantitative PCR for assessing mRNA expression

RNAs from 20 SACs and 22 hmMSI-H, including those from the training set, were extracted with the miRNeasy kit (ref: 217004, Qiagen) and used for validation by qPCR. The retrotranscriptase reaction was performed from a total of 1 μg of DNAseI-treated RNA using the DyNAmo cDNA synthesis Kit (ref: F470L) provided by Thermo Scientific (Rockford, IL). Five microlitres of 1:5 diluted cDNA was added to the qPCR reaction containing 12.5 μl 2X QuantiTect SYBR Green PCR Kit (ref:204145, Qiagen) and 300 nM of each primer in a total volume of 25 μl. qPCR was performed on a 7500F real-time PCR system by Applied Biosystems (Foster City, CA, USA) according to the instruction manual and following the standard protocol: 50 °C 2 min, 95 °C 10 min, 40 cycles of 95 °C 15 s, 60 °C 1 min and a melt curve stage consisting of 95 °C 15 s, 60 °C min, 95 °C 30 s and 60 °C 30 s. Primers were designed using primer3 software and sequences, and amplicon sizes are shown in Additional file 5: Table S3. The relative quantitation was done by the 2-ΔCt method using β-actin as the housekeeping gene.

### Immunohistochemistry

The validation subset consisted of 26 SAC and 21 hmMSI-H cases matched for gender, age and location, and a representative area of each tumour was selected by one of us (JGS). Whole 2.5-μm sections were stained with CD14 and HLADOA rabbit antibodies. Details on equipment, antigen retrieval conditions (buffer, pH, temperature, time) and incubation (temperature, time) for both antibodies are as follows: Bechmark Ultra Ventana, (CC1, basic, 95 °C, 56 min) and (overnight, room temperature). Antibody purveyor and type, code (clone) and antibody dilution were as follows: for CD14: Cell Marque, monoclonal, 760-4523 (EPR3653), 1:5, and for HLA-DOA: Sigma Aldrich, polyclonal, HPA045038, 1:200. Endogenous peroxidase activity was blocked using 0.5% H_2_O_2_ for 5 min. For visualization of the antigen, the sections were immersed in 3,3′-diaminobenzidine (DAB) and counterstained with Harris’ haematoxylin for 5 min. Following manufacturers´ recommendations sinusoidal histiocytes and mantle zone from a lymph node were used as positive controls for CD14 and HLA-DOA, respectively.

These markers were evaluated by considering a staining intensity in both the centre of the tumour and the invasive front (1 = none or weak staining, 2 = moderate, 3 = strong) and a staining area score (A < one third, B = between one and two thirds, C > two thirds) in a given area. For statistical analysis, both intensity and distribution were considered.

### Statistical analysis of validation data

For the analysis of quantification of methylated DNA sequences, the data correspond to a split-plot design with one between-subject factor defining six independent groups of cases (SAC, CC, hmMSI-H; tumoural and non-tumoural) and one within-subject factor (CpG sites) defining nine repeated measures for every case. Accordingly, we performed two ANOVA SPF-p-q. The first compared the means of the tumoural vs. non-tumoural groups in each of the nine different CpG sites and the second the means of the six different groups in these sites. For checking the relationship between methylation percentage and binary variables, the *t* test for independent samples and the Mann-Whitney’s *U* test were used. Statistical significance in the immunohistochemistry study was assessed using Pearson *χ*^2^ or Fisher’s exact test when indicated. Descriptive statistics were computed for real-time PCR. Statistical analysis was performed using the SPSS (Version 22, Chicago, IL) package.

## Additional files

Additional file 1: **Table S1.** Gene Ontology biological processes (BP) terms differentially enriched in the comparison SAC vs. hmMSI-H; 40 obtained using ClusterProfiler and 76 using FatiGo. (XLSX 25 kb)

Additional file 2: **Figure S1.** Terms of Gene Ontology Cellular Component and Molecular Functions enriched in the comparison between SAC and hmMSI-H for their methyloma profile. The scatterplot shows the terms which are globally differentially methylated between SAC and hmMSI-H after GO term redundancy reduction. The graph is represented in a two dimensional space derived by applying multidimensional scaling to a matrix of the GO terms’ semantic similarities [35]. (TIF 162 kb)

Additional file 3: **Table S2.** List of the 1540 differentially methylated genes between hmMSI-H and SAC. (XLSX 149 kb)

Additional file 4: Molecular functions of the 42 most differentially methylated genes. Source https://www.ncbi.nlm.nih.gov/gene. (DOCX 23 kb)

Additional file 5: **Table S3.** Primer sequences, amplicon sizes and location of CpGs evaluated in the study. (DOC 48 kb)

## Abbreviations

CC: Conventional carcinoma
CpG: Cytosine-phospho-guanine
CRC: Colorectal carcinoma
HLA: Human leukocyte antigen
hmMSI-H: Colorectal carcinoma showing typical molecular and histological features of MSI-H
IHC: Immunohistochemistry
MSI-H: High-grade microsatellite instability
MSS: Microsatellite stability
qPCR: Quantitative polymerase chain reaction
SAC: Serrated adenocarcinoma
SD: Standard deviation
WHO: World Health Organization
