# Supplementary figures and images for: Two histologically colorectal carcinomas subsets from the serrated pathway show different methylome signatures and diagnostic biomarkers
Source: Clin Epigenetics. 2018 Nov 9;10:141. doi: 10.1186/s13148-018-0571-3 (PMC6230233; doi:10.1186/s13148-018-0571-3)

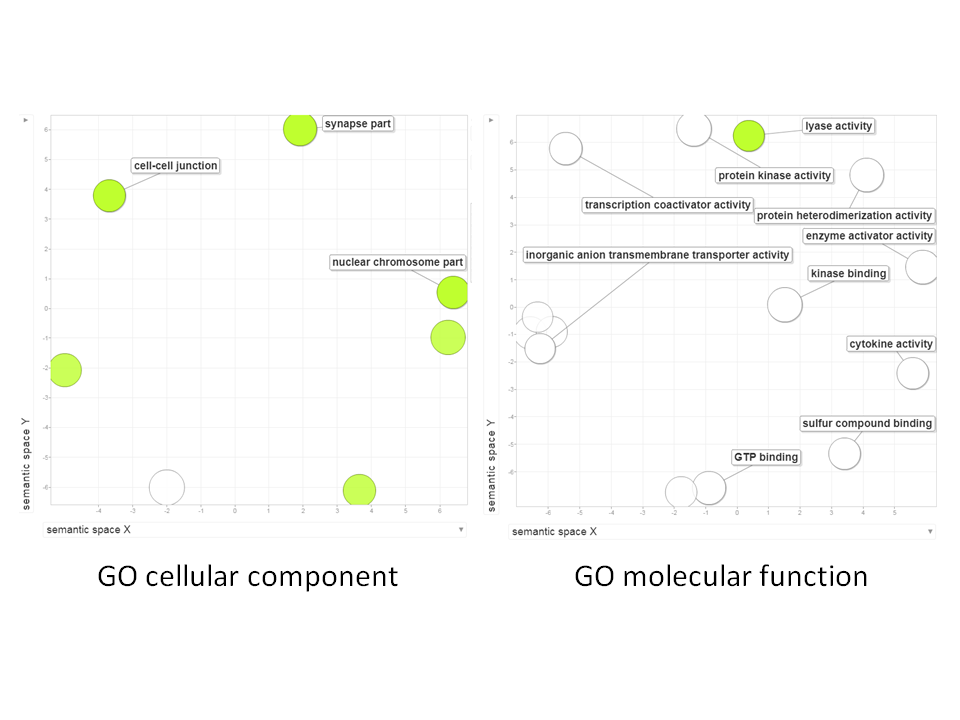

Supplement: Supplementary file 2 — Figure S1. Terms of Gene Ontology Cellular Component and Molecular Functions enriched in the comparison between SAC and hmMSI-H for their methyloma profile. The scatterplot shows the terms which are globally differentially methylated between SAC and hmMSI-H after GO term redundancy reduction. The graph is represented in a two dimensional space derived by applying multidimensional scaling to a matrix of the GO terms’ semantic similarities [35]. (TIF 162 kb) [file 13148_2018_571_MOESM2_ESM.tif]
