# Supplementary material for: Two histologically colorectal carcinomas subsets from the serrated pathway show different methylome signatures and diagnostic biomarkers
Source: Clin Epigenetics. 2018 Nov 9;10:141. doi: 10.1186/s13148-018-0571-3 (PMC6230233; doi:10.1186/s13148-018-0571-3)
Supplement: Supplementary file 4 — Molecular functions of the 42 most differentially methylated genes. Source https://www.ncbi.nlm.nih.gov/gene. (DOCX 23 kb) [file 13148_2018_571_MOESM4_ESM.docx]

**Supplemental material. S4.** Molecular functions associated with the 42 most differentially methylated genes. Source <https://www.ncbi.nlm.nih.gov/gene>. Accessed in Oct 2018:

*PDCD2L*

Protein which associates with arginine methyltransferase 3 (PRMT3) and contributes to the late maturation of 40S ribosomal subunits.

*FGFR2*

The protein encoded by this gene is a member of the fibroblast growth factor receptor family, where amino acid sequence is highly conserved between members and throughout evolution. FGFR family members differ from one another in their ligand affinities and tissue distribution. A full-length representative protein consists of an extracellular region, composed of three immunoglobulin-like domains, a single hydrophobic membrane-spanning segment and a cytoplasmic tyrosine kinase domain. The extracellular portion of the protein interacts with fibroblast growth factors, setting in motion a cascade of downstream signals, ultimately influencing mitogenesis and differentiation. This particular family member is a high-affinity receptor for acidic, basic and/or keratinocyte growth factor, depending on the isoform. Mutations in this gene are associated with Crouzon syndrome, Pfeiffer syndrome, Craniosynostosis, Apert syndrome, Jackson-Weiss syndrome, Beare-Stevenson cutis gyrata syndrome, Saethre-Chotzen syndrome, and syndromic craniosynostosis.

*CTSL2*

The protein encoded by this gene (cathepsin V), a member of the peptidase C1 family, is a lysosomal cysteine proteinase that may play an important role in corneal physiology. This gene is expressed in colorectal and breast carcinomas but not in normal colon, mammary gland, or peritumoral tissues, suggesting a possible role for this gene in tumor processes.

*TLR4*

The protein encoded by this gene is a member of the Toll-like receptor (TLR) family which plays a fundamental role in pathogen recognition and activation of innate immunity. TLRs are highly conserved from Drosophila to humans and share structural and functional similarities. They recognize pathogen-associated molecular patterns that are expressed on infectious agents, and mediate the production of cytokines necessary for the development of effective immunity. The various TLRs exhibit different patterns of expression. This receptor has been implicated in signal transduction events induced by lipopolysaccharide (LPS) found in most gram-negative bacteria. Mutations in this gene have been associated with differences in LPS responsiveness.

*ANKRD10*

Ankyrin repeat domain 10 mediates protein-protein interactions in very diverse families of proteins and it is expressed in spleen, endometrium and 25 other tissues, including colon.

*COPZ1*

This gene encodes a subunit of the cytoplasmic coatamer protein complex, which is involved in autophagy and intracellular protein trafficking. The coatomer protein complex is comprised of seven subunits and functions as the coat protein of coat protein complex (COP)I-vesicles.

*C17orf108*

*This gene is also known as* LYR motif containing 9 and some genetic variants has been associated with childhood asthma.

*CEP68*

Variants of the centrosomal protein 68 (CEP68) gene are associated with acute urticaria/angioedema induced by multiple non-steroidal anti-inflammatory drugs.

*ZNF652*

ZNF652 (zinc finger protein 652), specifically and functionally interacts with CBFA2T3 to repress transcription of genes involved in breast oncogenesis The ZNF652 and androgen receptor (AR) transcription factors are acting independently and it is proposed that the continued maintenance of expression of ZNF652 in AR positive prostate cancer cells results in a gene expression pattern that contributes to the relapse

*PCYOX1L*

Prenylcysteine oxidase 1 like (*PCYOX1L) seems to be involved platelet activation, signaling and aggregation, platelet degranulation and response to elevated platelet cytosolic Ca2+, organism-specific biosystem.*

*GALNT11*

pPlypeptide N-acetylgalactosaminyltransferase 11 (GALNT11) is expressed in B-CLL cells and normal T cell whereas little or no expression is found in normal B cells. GALNT11 expression is significantly associated with the mutational status of the immunoglobulin heavy chain variable region (IGHV)

*MICAL2*

Microtubule associated monooxygenase, calponin and LIM domain containing 2 (MICAL2) protein is a monooxygenase that enhances depolymerization of F-actin and is therefore involved in cytoskeletal dynamics. The encoded protein is a regulator of the SRF signaling pathway. Increased expression of this gene has been associated with cancer progression and metastasis.

*OSCP1*

organic solute carrier partner 1 (OSCP1) also knows as NOR1 is tumor suppressor gene involved in autophagy and apoptosis in nasopharyngeal carcinoma cells

*ACAD10*

This gene encodes a member of the acyl-CoA dehydrogenase family of enzymes (ACADs), which participate in the beta-oxidation of fatty acids in mitochondria. The encoded enzyme contains a hydrolase domain at the N-terminal portion, a serine/threonine protein kinase catlytic domain in the central region, and a conserved ACAD domain at the C-terminus.

*MELK*

Maternal embryonic leucine zipper kinase (MELK) is categorized as a member of AMP-activated protein kinase families. Various MELK-associated cellular and biological processes affect multiple stages of tumorigenesis

*HLA-DOA*

HLA-DOA belongs to the HLA class II alpha chain paralogues. HLA-DOA forms a heterodimer with HLA-DOB. The heterodimer, HLA-DO, is found in lysosomes in B cells and regulates HLA-DM-mediated peptide loading on MHC class II molecules. In comparison with classical HLA class II molecules, this gene exhibits very little sequence variation, especially at the protein level.

*KRT20*

The protein encoded by this gene is a member of the keratin family. The keratins are intermediate filament proteins responsible for the structural integrity of epithelial cells and are subdivided into cytokeratins and hair keratins. The type I cytokeratins consist of acidic proteins which are arranged in pairs of heterotypic keratin chains. This cytokeratin is a major cellular protein of mature enterocytes and goblet cells and is specifically expressed in the gastric and intestinal mucosa. The type I cytokeratin genes are clustered in a region of chromosome 17q12-q21.

*TMEM45B*

Transmembrane protein 45B (TMEM45B) is a member of the TMEM family of proteins and has been reported to be expressed abnormally in different kinds of human tumors.

*LOC285401*

*LOC285401 also known as* LINC00698 is a long intergenic non-protein coding RNA

*PPIL1*

This gene is a member of the cyclophilin family of peptidylprolyl isomerases (PPIases). The cyclophilins are a highly conserved, ubiquitous family, members of which play an important role in protein folding, immunosuppression by cyclosporin A, and infection of HIV-1 virions. Based on similarity to other PPIases, this protein could accelerate the folding of proteins and might catalyze the cis-trans isomerization of proline imidic peptide bonds in oligopeptides.

*CA13*

The encoded protein is a member of carbonic anhydrases (CAs), a family of zinc metalloenzymes. As catalysts of the reversible hydration of carbon dioxide, these enzymes participate in a variety of biologic processes, including respiration, calcification, acid-base balance, bone resorption, and the formation of aqueous humor, cerebrospinal fluid, saliva, and gastric acid.

*BCL7C*

This gene is identified by the similarity of its product to the N-terminal region of BCL7A protein. The BCL7A protein is encoded by the gene known to be directly involved in a three-way gene translocation in a Burkitt lymphoma cell line. The function of this gene has not yet been determined. Two transcript variants encoding different isoforms have been found for this gene.

*CD14*

The protein encoded by this gene is a surface antigen that is preferentially expressed on monocytes/macrophages. It cooperates with other proteins to mediate the innate immune response to bacterial lipopolysaccharide. Alternative splicing results in multiple transcript variants encoding the same protein.

*PLA2G4C*

This gene encodes a protein which is a member of the phospholipase A2 enzyme family which hydrolyzes glycerophospholipids to produce free fatty acids and lysophospholipids, both of which serve as precursors in the production of signaling molecules. The encoded protein has been shown to be a calcium-independent and membrane bound enzyme.

*LY6G6D*

LY6G6D belongs to a cluster of leukocyte antigen-6 (LY6) genes located in the major histocompatibility complex (MHC) class III region on chromosome 6. Members of the LY6 superfamily typically contain 70 to 80 amino acids, including 8 to 10 cysteines. Most LY6 proteins are attached to the cell surface by a glycosylphosphatidylinositol (GPI) anchor that is directly involved in signal transduction.

*TCF7L2*

This gene encodes a high mobility group (HMG) box-containing transcription factor that plays a key role in the Wnt signaling pathway. The protein has been implicated in blood glucose homeostasis. Genetic variants of this gene are associated with increased risk of type 2 diabetes. Several transcript variants encoding multiple different isoforms have been found for this gene.

*RP11-165H20.1*

This transcript is a CHIA-like pseudogene. CHIA encode for acidic mammalian chitinase (AMCase), an enzyme implicated in the pathology of asthma and capable of chitin cleavage at a low pH optimum

*CHD6*

This gene encodes a member of the SNF2/RAD54 helicase protein family. The encoded protein contains two chromodomains, a helicase domain, and an ATPase domain. Several multi-subunit protein complexes remodel chromatin to allow patterns of cell type-specific gene expression, and the encoded protein is thought to be a core member of one or more of these chromatin remodeling complexes. The encoded protein may function as a transcriptional repressor and is involved in the cellular repression of influenza virus replication.

*PARN*

The protein encoded by this gene is a 3'-exoribonuclease, with similarity to the RNase D family of 3'-exonucleases. It prefers poly(A) as the substrate, hence, efficiently degrades poly(A) tails of mRNAs. Exonucleolytic degradation of the poly(A) tail is often the first step in the decay of eukaryotic mRNAs. This protein is also involved in silencing of certain maternal mRNAs during oocyte maturation and early embryonic development, as well as in nonsense-mediated decay (NMD) of mRNAs that contain premature stop codons. Alternatively spliced transcript variants encoding different isoforms have been found for this gene.

*KCNK15*

This gene encodes one of the members of the superfamily of potassium channel proteins containing two pore-forming P domains. The product of this gene has not been shown to be a functional channel, however, it may require other non-pore-forming proteins for activity.

*TMEM209*

This gene encondes the transmembrane protein 209, also known as NET31. It is an integral nuclear envelope protein. TMEM209 expression is normally limited to testis, but it is was widely expressed in lung cancer. Ectopic overexpression of TMEM209 promoted cell growth, whereas TMEM209 attenuation was sufficient to block growth.

*GOLM1*

The Golgi complex plays a key role in the sorting and modification of proteins exported from the endoplasmic reticulum. The protein encoded by this gene is a type II Golgi transmembrane protein. It processes proteins synthesized in the rough endoplasmic reticulum and assists in the transport of protein cargo through the Golgi apparatus. The expression of this gene has been observed to be upregulated in response to viral infection.

*ARRDC1*

*This gene encodes Α-arrestin 1 (*ARRDC1) a protein that promotes non-activated Notch receptor degradation, thus acting as a negative regulator of Notch signaling.

*TRIM21*

This gene encodes a member of the tripartite motif (TRIM) family. The TRIM motif includes three zinc-binding domains, a RING, a B-box type 1 and a B-box type 2, and a coiled-coil region. The encoded protein is part of the RoSSA ribonucleoprotein, which includes a single polypeptide and one of four small RNA molecules. The RoSSA particle localizes to both the cytoplasm and the nucleus. RoSSA interacts with autoantigens in patients with Sjogren syndrome and systemic lupus erythematosus.

*PHAX*

This gene encodes the phosphorylated adaptor for nuclear export (PHAX) which is required for nuclear export of snRNAs in metazoans and also involved in the intranuclear transport of small nucleolar RNAs to Cajal bodies.

*NMT2*

This gene encodes one of two N-myristoyltransferase proteins. N-terminal myristoylation is a lipid modification that is involved in regulating the function and localization of signaling proteins. The encoded protein catalyzes the addition of a myristoyl group to the N-terminal glycine residue of many signaling proteins, including the human immunodeficiency virus type 1 (HIV-1) proteins, Gag and Nef.

*ATP12A*

The protein encoded by this gene belongs to the family of P-type cation transport ATPases. This gene encodes a catalytic subunit of the ouabain-sensitive H+/K+ -ATPase that catalyzes the hydrolysis of ATP coupled with the exchange of H(+) and K(+) ions across the plasma membrane. It is also responsible for potassium absorption in various tissues. Two transcript variants encoding different isoforms have been found for this gene.

*ARHGAP30*

Rho GTPase activating protein 30 (*ARHGAP30*) act as a Wrch-1 interactor, a atypical Rho GTPase which has roles in cell migration, focal adhesion dissolution, stress fibre break down and tight junction heterogeneity. ARHGAP30 is related to the Cdc42- and Rac1-specific RhoGAP CdGAP, which was likewise found to bind Wrch-1.

*WWP1*

WW domain-containing proteins are found in all eukaryotes and play an important role in the regulation of a wide variety of cellular functions such as protein degradation, transcription, and RNA splicing. This gene encodes a protein which contains 4 tandem WW domains and a HECT (homologous to the E6-associated protein carboxyl terminus) domain. The encoded protein belongs to a family of NEDD4-like proteins, which are E3 ubiquitin-ligase molecules and regulate key trafficking decisions, including targeting of proteins to proteosomes or lysosomes.

*GNAI3*

Guanine nucleotide-binding proteins (G proteins) are involved as modulators or transducers in various transmembrane signaling pathways. G proteins are composed of 3 units: alpha, beta and gamma. This gene encodes an alpha subunit and belongs to the G-alpha family. Mutation in this gene, resulting in a gly40-to-arg substitution, is associated with auriculocondylar syndrome, and shown to affect downstream targets in the G protein-coupled endothelin receptor pathway.

*CD48*

This gene encodes a member of the CD2 subfamily of immunoglobulin-like receptors which includes SLAM (signaling lymphocyte activation molecules) proteins. The encoded protein is found on the surface of lymphocytes and other immune cells, dendritic cells and endothelial cells, and participates in activation and differentiation pathways in these cells. The encoded protein does not have a transmembrane domain, however, but is held at the cell surface by a GPI anchor via a C-terminal domain which maybe cleaved to yield a soluble form of the receptor.

*EXPH5*

The protein encoded by this gene is a member of the synaptotagmin-like protein (Slp) family lacking a C2 domain. It contains an N-terminal synaptotagmin-like homology domain (SHD), and is a ras-related protein Rab-27B effector protein. This protein is thought to be involved in exosome secretion and intracellular vesicle trafficking. Reduced expression of this gene results in keratin filament defects. Mutations in this gene have been associated with some cases of epidermolysis bullosa, an inherited skin fragility disorder. Alternative splicing results in multiple transcript variants encoding different isoforms.
