# Supplementary material for: Two histologically colorectal carcinomas subsets from the serrated pathway show different methylome signatures and diagnostic biomarkers
Source: Clin Epigenetics. 2018 Nov 9;10:141. doi: 10.1186/s13148-018-0571-3 (PMC6230233; doi:10.1186/s13148-018-0571-3)
Supplement: Supplementary file 5 — Table S3. Primer sequences, amplicon sizes and location of CpGs evaluated in the study. (DOC 48 kb) [file 13148_2018_571_MOESM5_ESM.doc]

**Supplemental material S5**

Sequence and procedure information for the different molecular techniques carried out.

**1.** Pyrosequencing for the relative quantitation of CpG methylation in *CD14* and *HLA-DOA* genes

| **Gene** | **Primer name** | **Primer sequence 5´-3´** | **Annealing**  **Temperature** | **Amplicon size (bp)** |
| --- | --- | --- | --- | --- |
| *CD14* | CD14pF | GGGTTTATAGAGGAGGGAATTGAAT | 53ºC | 194 |
|  | bCD14pR | [Btn]CAAAACCCTACACTCACCATAAT |
|  | CD14pseq | GGATTATATAAATTGTTAGAGGTAG | | |
|  | Sequence to analyse | TYGAAGAGTTTATAAGTGTGAAGTTTGGAAGTTGGTGGGTGTYGT | | |
|  | Unconverted  amplicon sequence | GGGTTCACAGAGGAGGGAACTGAATGACATCCCAGGATTACATAAACTGT  CAGAGGCAGC**CG**AAGAGTTCACAAGTGTGAAGCCTGGAAGCCGGCGGGT  GC**CG**CTGTGTAGGAAAGAAGCTAAAGCACTTCCAGAGCCTGTCCGGAGCT  CAGAGGTTCGGAAGACTTATCGACCATGGTGAGTGTAGGGTCTTG | | |
| *HLA-DOA* | HLA-DOAp F | GGGGAGTATGTTGAAAATATAGGT | 59ºC | 211 |
|  | HLA-DOAp R | [Btn]AAAATCTAACCAAATTAAACCATATATCA |
|  | HLA-DOApseq | GTAAAGAGGAAATTTAGGAA | | |
|  | Sequence to analyse | TAYGTATTATAAYGAATTTTTTAGGTGATTTTTATTTGGAATTAGAGATTYGT | | |
|  | Unconverted  amplicon sequence | GGGGAGCATGCTGAAAACACAGGTTCCCTGGCCCTGCCTTGGAAAGGCTA  ATCTAACCCATTAAGTAAAGAGGAAACTCAGGAACA**CG**TATTATAA**CG**AAC  CCCCCAGGTGATTCTCATTTGGAACCAGAGACC**CG**CATGATTTCCTAGCTC  CCTCCAAGTTGAAAATCATCGATTTCATGGTCTTTGACACATGGCCTAATTT  GGTTAGA | | |

[Btn]: Biotin labelled

T: Converted unmethylated citosines after bisulfite treatment of direct sequences.

Y: Ambiguity code for C/T

**CG:** CpG sites studied

Sequencing primers

PCR primer areas

**2.** Quantitative PCR primers for the quantitation of mRNA expression of *CD14* and *HLA-DOA* genes using *β-actin* as a house-keeping gene.

| Primer name | Primer sequence 5´-3´ | Annealing  temperature | Amplicon size (bp) |
| --- | --- | --- | --- |
| CD14qF | GAGCTCAGAGGTTCGGAAGA | 60 | 93 |
| CD14qR | CGTGGTCGCAGAGACGTG |
| HLA-DOAqF | GGCACCGTCCTCATCATC | 60 | 109 |
| HLA-DOAqR | ACAAACCCATGAGGATCTGC |
| B-ACTIN-RTF | GAGCTACGAGCTGCCTGACG | 60 | 122 |
| B-ACTIN-RTR | GTAGTTTCGTGGATGCCACAG |
